# Personality typologies of smokers and excessive drinkers: a cross-sectional survey of respondents in the BBC Lab UK Study

**DOI:** 10.12688/f1000research.86670.2

**Published:** 2023-03-02

**Authors:** Olga Perski, Astrid Nikiel, Jamie Brown, Lion Shahab

**Affiliations:** 1 Department of Behavioural Science and Health, University College London, London, UK

**Keywords:** tobacco smoking; excessive alcohol consumption; personality typologies; cross-sectional survey

## Abstract

**Background:** Several personality traits have been linked to addictive behaviours, including smoking and excessive drinking. We hypothesised that the combination of low conscientiousness, high extraversion and high neuroticism would be synergistically associated with smoking, excessive drinking and both behaviours combined.

**Methods:** Respondents aged 16+ years ( *N*=363,454) were surveyed between 2009-2013 as part of the BBC Lab UK Study, with no restrictions on geographical location. Respondents provided information about sociodemographic characteristics, personality traits, and smoking and alcohol consumption. A series of multivariable logistic regression analyses were conducted.

**Results:** No significant three-way but significant two-way interactive effects were observed. The association of high extraversion with smoking was more pronounced in those with high (vs. low) conscientiousness (OR _adj_=1.51, 95% CI=1.46, 1.56, *p*<.001; OR _adj_=1.38, 95% CI=1.35, 1.42, *p*<.001). The association of high extraversion with excessive drinking was more pronounced in those with low (vs. high) conscientiousness (OR _adj_=1.70, 95% CI=1.67, 1.74, *p*<.001; OR _adj_=1.60, 95% CI=1.56, 1.63, *p*<.001). The association of high extraversion with both behaviours combined was more pronounced in those with high (vs. low) conscientiousness (OR _adj_=1.74, 95% CI=1.65, 1.83, *p*<.001; OR _adj_=1.62, 95% CI= 1.56, 1.68, *p*<.001). Results remained largely robust in sensitivity analyses.

**Conclusions:** In a large international survey, we identified two-way ‘personality typologies’ that are associated with greater odds of smoking, excessive drinking and both behaviours combined. The results may be useful for the tailoring of behaviour change interventions to at-risk individuals.

## Introduction

Cigarette smoking and excessive alcohol consumption are two of the most serious public health problems globally ( Stanaway *et al.*, 2018). Each year, up to eight million people die of a smoking-related disease ( World Health Organisation, 2021). Excessive alcohol consumption resulted in three million global deaths in 2016, the majority of which were due to injuries or digestive diseases ( World Health Organisation, 2018a). Excessive alcohol consumption is implicated in substantial costs to the economy through lost productivity, crime and healthcare costs ( Gowing *et al.*, 2015; Lim *et al.*, 2012). Differences in human personality can be accounted for by a limited number of dimensions or traits ( Cattell, 1973). The “Big Five” model is a widely applied taxonomy which proposes that personality is underpinned by five major (and intentionally broad) dimensions: openness to experience (i.e. the tendency to be curious and excitable), conscientiousness (i.e. the tendency to be organised and deliberative), extraversion (i.e. the tendency to be sociable and outgoing), agreeableness (i.e. the tendency to be sympathetic and warm) and neuroticism (i.e. the tendency to be self-conscious and moody) ( Costa Jr & McCrae, 1992). Several personality traits have been linked to addictive behaviours in general, and with smoking and excessive alcohol consumption in particular (outlined in detail below). To the authors’ knowledge, no study to date has explored whether particular evidence-informed combinations of personality traits (i.e. ‘personality typologies’) are synergistically associated with greater odds of being a current smoker, excessive drinker or both. We aimed to explore this in a large, cross-sectional sample of respondents from the international British Broadcasting Corporation (BBC) Lab UK Study.

### Openness to experience

Evidence for associations between openness to experience and smoking and excessive drinking is mixed: while some studies have found a positive association of openness with excessive drinking ( Martin *et al.*, 2015), others have found a negative or non-significant association with smoking ( Jokela *et al.*, 2018; McCann, 2010) or excessive drinking ( Luchetti *et al.*, 2018; Scaife & Duka, 2009).

### Conscientiousness

As conscientiousness comprises facets of self-discipline and deliberation, it is plausible that the association between this trait and smoking and drinking is mediated by behavioural mechanisms associated with self-discipline (e.g. reduced exposure to others who smoke or drink, increased ability to inhibit prepotent responses to smoking or alcohol-related environmental cues). Several cross-sectional and longitudinal studies of representative and non-representative samples indicate that low conscientiousness is associated with increased risk of smoking ( Bogg & Roberts, 2004; Hagger-Johnson *et al.*, 2012; Hampson *et al*., 2006; Hong & Paunonen, 2009; Jokela *et al.*, 2018; Raynor & Levine, 2010; Terracciano & Costa Jr, 2004; Welch & Poulton, 2009) and excessive drinking ( Adan *et al.*, 2017; Bogg & Roberts, 2004; Hagger-Johnson *et al.*, 2012; Hakulinen *et al.*, 2015; Ibáñez *et al.*, 2015; Jokela *et al.*, 2018; Kotov *et al.*, 2010; Luchetti *et al.*, 2018; Malouff *et al.*, 2007; Raynor & Levine, 2010; Ruiz *et al.*, 2010).

### Extraversion

As extraversion comprises facets of sociability and sensation seeking, it has been suggested that the association between this trait and smoking and drinking might be mediated by behavioural or biological mechanisms associated with sociability (e.g. greater exposure to others who smoke or drink) or sensation seeking (e.g. experiencing a proportionately greater reinforcing, as opposed to aversive, effect of nicotine or ethanol) ( Munafò *et al.*, 2007). In cross-sectional and prospective studies, high extraversion is associated with increased odds of smoking ( Jokela *et al.*, 2018; Munafò *et al.*, 2007; Raynor & Levine, 2010) and excessive drinking ( Adan *et al.*, 2017; Cheng & Furnham, 2013; Hakulinen *et al.*, 2015; Ibáñez *et al.*, 2015; Jokela *et al.*, 2018; Luchetti *et al.*, 2018; Raynor & Levine, 2010).

### Agreeableness

Evidence for an association between agreeableness and smoking and excessive drinking is mixed: while some studies have found a positive association of agreeableness with excessive drinking ( Whelan *et al.*, 2014), others have found a negative association with smoking ( Hampson *et al.*, 2006; Hong & Paunonen, 2009; Terracciano & Costa Jr, 2004) or excessive drinking ( Cheng & Furnham, 2013; Hong & Paunonen, 2009; Ibáñez *et al.*, 2015; Jokela *et al.*, 2018; Luchetti *et al.*, 2018; Malouff *et al.*, 2007).

### Neuroticism

As neuroticism comprises facets of emotional instability and negative affect, it has been proposed that individuals who score highly on neuroticism may self-medicate with cigarettes or alcohol to reduce anxiety and enhance low mood ( Munafò *et al.*, 2007). Results from cross-sectional and longitudinal studies indicate that individuals scoring high on neuroticism have increased odds of being a smoker ( Munafò *et al.*, 2007; Terracciano & Costa Jr, 2004; Welch & Poulton, 2009) and excessive drinker ( Adan *et al.*, 2017; Jokela *et al.*, 2018; Kotov *et al.*, 2010; Luchetti *et al.*, 2018; Malouff *et al.*, 2007; Ruiz *et al.*, 2010). However, in a sample of university students, low neuroticism was associated with being categorised as a heavy drinker ( Lac & Donaldson, 2016).

### Evidence-informed ‘personality typologies’

Although several studies have examined the independent associations between the Big Five personality traits and smoking/excessive drinking, few studies have assessed their synergistic (or interactive) effects, henceforth referred to as ‘personality typologies’. Previous research has found an interactive effect of low conscientiousness and low agreeableness ( Hong & Paunonen, 2009) and of low conscientiousness and high neuroticism ( Terracciano & Costa Jr, 2004) on smoking. Based on the evidence reviewed above, we hypothesised that the combination of (i.e. the three-way interaction between) low conscientiousness, high extraversion and high neuroticism would be synergistically associated with smoking, excessive drinking and the combination of both behaviours. As limited research has been conducted relating to openness to experience and agreeableness, with available studies providing mixed evidence of the associations between these personality traits and addictive behaviours, we were unable to generate a directional hypothesis for these traits.

### Gender, age and socioeconomic status

Evidence suggests that personality traits ( Feingold, 1994), smoking ( Peters *et al.*, 2014) and alcohol consumption ( Wilsnack *et al.*, 2000) vary by sex/gender. There is also evidence to suggest that personality may change over time, with average declines in neuroticism, extraversion and openness, and increases in agreeableness and conscientiousness, over the life span ( McCrae *et al*., 1999). Moreover, low socioeconomic status is associated with both smoking ( Casetta *et al.*, 2017) and excessive drinking ( Beard *et al.*, 2019) and the prevalence of both behaviours vary by country ( World Health Organisation, 2018b, 2019). We therefore included sex, age, socioeconomic status (measured by education) and country of residence as covariates in our analyses.

Specifically, this study aimed to address the following research questions in a large, cross-sectional sample:

1. Is the combination of low conscientiousness, high extraversion and high neuroticism synergistically associated with current smoking, without and with adjustment for sex, age, education and country of residence?
2. Is the combination of low conscientiousness, high extraversion and high neuroticism synergistically associated with excessive drinking, without and with adjustment for sex, age, education and country of residence?
3. Is the combination of low conscientiousness, high extraversion and high neuroticism synergistically associated with the combination of current smoking and excessive drinking, without and with adjustment for sex, age, education and country of residence?

## Methods

### Study design and setting

This was a correlational study involving cross-sectional data. The STROBE guidelines were used in the design and reporting of this study ( Elm *et al.*, 2007). The study protocol and analysis plan were pre-registered on the Open Science Framework (OSF; osf.io/5h9sj). A series of open access online surveys were hosted by the BBC Lab UK Study website between 2009 and 2013. Anyone able to access the website could take part. The pre-registered protocol specified that only respondents from the UK would be included; however, we subsequently decided to include respondents irrespective of their country of residence.

### Inclusion criteria

Respondents who were aged 16+ years were included.

### Sample recruitment

Interested respondents were invited to take part in open access experiments and surveys via the BBC Lab UK website. The survey was advertised and promoted via the BBC website, radio programmes and television shows. This was a citizen science project with data being collected by members of the general public in collaboration with scientists. As such, participants were not reimbursed for their time.

### Ethical approval

This study involved secondary analyses of fully anonymised data obtained from the BBC Lab UK Study. Hence, ethical approval was not sought. Respondents were told that by clicking on the link to proceed to the survey, they were providing consent to participate. Initiating the survey was used as a record of consent.

### Measures

*Outcome variables*

The outcome variables were current smoking (no vs. yes), excessive drinking (no vs. yes) and the combination of both behaviours (no vs. yes). Current smoking was assessed by the following two items: “Have you ever smoked cigarettes daily, that is, at least one cigarette every day for 30 days?” and “During the past 30 days, on average how many cigarettes did you smoke per day?”. Respondents indicating that they had ever smoked cigarettes daily and any cigarettes in the past 30 days were coded as a ‘current smoker’. Excessive drinking was assessed with the following item: “During the past 30 days, on how many days did you have 5 or more drinks of alcohol in a row, that is, within a couple of hours?” Response options were: 1) 0 days, 2) 1 day, 3) 2 days, 4) 3 to 5 days, 5) 6 to 9 days, 6) 10 to 19 days, 7) 20 to 29 days and 8) all 30 days. Respondents were dichotomised into ‘low/moderate drinkers’ (response options 1-2) and ‘excessive drinkers’ (response options 3-8); this deviated from the pre-registered analysis plan, in which we had specified that response options 2-8 would be categorised as ‘excessive drinkers’. This item broadly corresponds to the third item on the validated Alcohol Use Disorders Identification Test-Consumption (AUDIT-C) scale ( Frank *et al.*, 2008) and single-item measures of binge drinking ( Wechsler *et al.*, 1995). This deviation was based on a better fit with the validated measure (AUDIT-C). The original categorisation would have overestimated excessive drinking. Respondents categorised as both a ‘current smoker’ and ‘excessive drinker’ received a score of 1 on the clustering variable, with those categorised as either or neither receiving a score of 0.

*Explanatory variables*

The explanatory variables were extraversion, conscientiousness and neuroticism, measured with the Big Five Inventory ( John *et al.*, 1991). Raw scores were transformed by the BBC Lab UK Study team into a percentage of maximum possible (POMP) score ranging from 0 to 100. As population norms for the Big Five Inventory are lacking, we relied on local norms to categorise responses (low vs. high) using the median split. In a planned sensitivity analysis (pre-specified on the OSF; osf.io/5h9sj), we categorised those scoring 1 standard deviation (SD) above the mean POMP score for each trait as ‘high’ (or 1 SD below the mean as ‘low’ for conscientiousness) vs. all others. In an unplanned sensitivity analysis, we categorised those scoring 1 SD above the mean POMP score for each trait as ‘high’ and those scoring 1 SD below the mean as ‘low’, excluding participants falling within 1 SD either side. This alternative cut-off was selected based on the assumption that any effect is more likely to be detected at the extreme ends of the personality scales and that such a cut-off may also be more clinically useful.

*Covariates*

Covariates were sex (male, female), age (continuous) and education, measured by combining responses to the following two items: “What is your highest level of formal schooling?” and “If you are still in education, what is the highest level of education you expect to obtain?”. For respondents indicating that they were still in education, responses to the second item were used. Response options were: 1) Did not complete GCSE/CSE/O-Levels, 2) Completed GCSE/CSE/O-Levels, 3) Completed post-16 vocational course, 4) A-Levels, 5) Undergraduate degree, 6) Postgraduate degree. Responses were then categorised into ‘no post-16 qualifications’ (response options 1-2), ‘post-16 qualifications’ (response options 3-4) and ‘higher education’ (response options 5-6). The age variable was capped at 100 years, with responses >100 coded as missing. Respondents were also asked to indicate their country of residence.

### Data analysis

Data were analysed in RStudio v.3.5.2. Respondents with missing data on any of the variables of interest were excluded from the analyses.

To address the research questions, multivariable logistic regression analyses were conducted for each of the three outcome variables, including each personality trait, the component two-way interaction effects of the personality traits and the three-way interaction effect of the personality traits, without and with adjustment for all covariates.

*Bayes factors*

Planned further analyses involved the calculation of Bayes Factors (BFs) using an online calculator to examine whether non-significant associations could best be characterised as evidence of no effect or whether the data were insensitive to detect an effect. In the limited research on synergistic associations between two personality traits and smoking/excessive drinking, small effects have been identified ( Terracciano & Costa Jr, 2004). At the same time, meta-analyses and syntheses of data from multiple cohort studies suggest that independent associations between personality traits and smoking/excessive drinking also tend to be small at approximately OR = 1.36 (extraversion and smoking) and OR = 1.2 (neuroticism and excessive drinking) ( Hakulinen *et al.*, 2015; Munafò *et al.*, 2007). For a synergistic association (i.e. a three-way interaction) to be considered meaningful, we proposed that the effect needed to be at least similar to those observed for the independent associations between the Big Five traits and smoking/excessive drinking. We therefore set the expected effect sizes to OR = 1.4. The alternative hypothesis was conservatively represented by a half-normal distribution. BFs of ≥3 can be interpreted as substantial evidence for the alternative hypothesis (and against the null), while BFs of ≤1/3 can be interpreted as evidence for the null hypothesis. BFs between 1/3 and 3 suggest that the data are insensitive to distinguish the alternative hypothesis from the null ( Dienes, 2011).

## Results

A total of 588,014 respondents completed the survey, with 363,454 (61.8%) respondents included in the analytic sample (see Table 1). Compared with the overall sample, a nominally greater proportion of those included in the analytic sample had a higher education, resided in the UK, were a current smoker, were an excessive drinker and were a combined smoker and excessive drinker.

**Table 1.** Respondents’ sociodemographic, smoking, drinking and personality characteristics.

	Overall ( *N* = 588,014)	Excluded ( *N* = 224,560)	Included ( *N* = 363,454)
**Sex, n (%)**			
Male	196,053 (33.3%)	70,106 (31.2%)	125,947 (34.7%)
Female	344,931 (58.7%)	107,424 (47.8%)	237,507 (65.3%)
Missing	47,030 (8%)	47,030 (20.9%)	0 (0%)
**Age**			
Mean ( *SD*)	33.9 ( *14.1*)	34.1 ( *15.7*)	33.8 ( *13.0*)
Missing	6,436 (%)	6,436 (%)	0 (0%)
**Education, n (%)**			
No post-16 qualifications	93,386 (16%)	36,088 (16%)	57,298 (16%)
Post-16 qualifications	97,832 (17%)	30,148 (13%)	67,684 (19%)
Higher education	315,695 (54%)	77,223 (34%)	238,472 (66%)
Missing	81,101 (14%)	81,101 (36%)	0 (0%)
**Country, n (%)**			
United Kingdom	502,495 (85%)	186,813 (83%)	315,682 (87%)
United States	25,302 (4.3%)	10,482 (4.7%)	14,820 (4.1%)
Ireland	8,179 (1.4%)	2,824 (1.3%)	5,355 (1.5%)
Canada	4,622 (0.8%)	1,788 (0.8%)	2,834 (0.8%)
Australia	3,936 (0.7%)	1,502 (0.7%)	2,434 (0.7%)
India	3,895 (0.7%)	1,578 (0.7%)	2,317 (0.6%)
New Zealand	2,233 (0.4%)	653 (0.3%)	1,580 (0.4%)
The Netherlands	2,142 (0.4%)	792 (0.4%)	1,350 (0.4%)
Other	28,604 (4.9%)	11,522 (5.1%)	17,082 (4.7%)
Missing	6,606 (1.1%)	6,606 (2.9%)	0 (0%)
**Smoker, n (%)**			
No	325,325 (55.3%)	3,795 (1.7%)	321,530 (88.5%)
Yes	42,534 (7.2%)	610 (0.3%)	41,924 (11.5%)
Missing	220,155 (37.4%)	220,155 (98%)	0 (0%)
**Excessive drinker, n (%)**			
No	308,281 (52.4%)	56,355 (25.1%)	251,926 (69.3%)
Yes	155,249 (26.4%)	43,721 (19.5%)	111,528 (30.7%)
Missing	124,484 (21.2%)	124,484 (55.4%)	0 (0%)
**Combined smoker and excessive drinker, n (%)**			
No	346,154 (58.9%)	4,115 (1.8%)	342,039 (94.1%)
Yes	21,705 (3.7%)	290 (0.1%)	21,415 (5.9%)
Missing	220,155 (37.4%)	220,155 (98%)	0 (0%)
**Extraversion, n (%)**			
Low	232,603 (39.6%)	56,775 (25.3%)	175,828 (48%)
High	260,456 (44.3%)	72,830 (32.4%)	187,626 (52%)
Missing	94,955 (16.1%)	94,955 (42.3%)	0 (0%)
**Conscientiousness, n (%)**			
Low	236,693 (40.3%)	66,471 (29.6%)	170,222 (47%)
High	256,366 (43.6%)	63,134 (28.1%)	193,232 (53%)
Missing	94,955 (16.1%)	94,955 (42.3%)	0 (0%)
**Neuroticism, n (%)**			
Low	236,572 (40.2%)	61,422 (27.4%)	175,150 (48%)
High	256,487 (43.6%)	68,183 (30.4%)	188,304 (52%)
Missing	94,955 (16.1%)	94,955 (42.3%)	0 (0%)

### Is the combination of low conscientiousness, high extraversion and high neuroticism synergistically associated with a) current smoking, b) excessive drinking or c) both behaviours combined?

No significant three-way interactions were observed (see Table 2). However, significant two-way interactive effects of high extraversion and low conscientiousness on smoking, excessive drinking and both behaviours combined were observed, with associations remaining robust in the covariate adjusted model. These interactions were further explored in stratified analyses. In addition, significant two-way interactive effects of low conscientiousness and high neuroticism on smoking and excessive drinking were observed, but these associations were no longer significant following adjustment for covariates.

**Table 2.** Multivariable logistic regression analyses estimating the association of the evidence-informed personality typologies with a) smoking, b) excessive drinking and c) both behaviours combined, without (OR) and with (OR _adj_) adjustment for covariates.

	Smoking			Excessive drinking			Combined		
	OR	95% CI	p-value	OR	95% CI	*p*-value	OR	95% CI	*p*-value
**Extraversion**									
Low (ref)	—	—		—	—		—	—	
High	1.61	1.53, 1.69	**<.001**	1.54	1.49, 1.58	**<.001**	1.89	1.75, 2.04	**<.001**
**Conscientiousness**									
High (ref)	—	—		—	—		—	—	
Low	1.82	1.72, 1.93	**<.001**	1.67	1.61, 1.73	**<.001**	2.48	2.28, 2.70	**<.001**
**Neuroticism**									
Low (ref)	—	—		—	—		—	—	
High	1.22	1.16, 1.29	**<.001**	0.98	0.95, 1.01	.2	1.27	1.16, 1.38	**<.001**
**Extraversion × Conscientiousness**									
High * Low	0.88	0.83, 0.95	**<.001**	1.05	1.01, 1.10	**.027**	0.85	0.77, 0.94	**.001**
**Extraversion × Neuroticism**									
High * High	0.97	0.91, 1.04	.4	1.02	0.98, 1.07	.3	0.95	0.86, 1.05	.3
**Conscientiousness × Neuroticism**									
Low * High	1.09	1.01, 1.16	**.021**	0.91	0.87, 0.96	**<.001**	0.96	0.87, 1.06	.4
**Extraversion × Conscientiousness × Neuroticism**									
High * Low * High	1.05	0.96, 1.15	.3	0.99	0.93, 1.05	.8	1.13	0.99, 1.28	.062

	Smoking			Excessive drinking			Combined		
	OR _adj_	95% CI	p-value	OR _adj_	95% CI	*p*-value	OR _adj_	95% CI	*p*-value
**Extraversion**									
Low (ref)	—	—		—	—		—	—	
High	1.58	1.51, 1.66	**<.001**	1.60	1.55, 1.65	**<.001**	1.87	1.73, 2.01	**<.001**
**Conscientiousness**									
High (ref)	—	—		—	—		—	—	
Low	1.64	1.54, 1.73	**<.001**	1.37	1.32, 1.42	**<.001**	2.02	1.86, 2.20	**<.001**
**Neuroticism**									
Low (ref)	—	—		—	—		—	—	
High	1.21	1.15, 1.28	**<.001**	1.03	1.00, 1.07	.054	1.26	1.16, 1.38	**<.001**
**Extraversion × Conscientiousness**									
High * Low	0.91	0.85, 0.97	**.006**	1.07	1.02, 1.12	**.005**	0.87	0.79, 0.96	**.007**
**Extraversion × Neuroticism**									
High * High	0.99	0.92, 1.06	.8	1.01	0.97, 1.06	.6	0.96	0.87, 1.07	.5
**Conscientiousness × Neuroticism**									
Low * High	1.07	1.00, 1.15	.064	0.96	0.92, 1.01	.081	0.98	0.88, 1.08	.6
**Extraversion × Conscientiousness × Neuroticism**									
High * Low * High	1.04	0.95, 1.14	.3	0.99	0.94, 1.06	.9	1.12	0.99, 1.28	.075
**Age**	0.99	0.99, 0.99	**<.001**	0.97	0.97, 0.98	**<.001**	0.97	0.97, 0.97	**<.001**
**Sex**									
Male	—	—		—	—		—	—	
Female	0.90	0.88, 0.92	**<.001**	0.50	0.49, 0.51	**<.001**	0.71	0.69, 0.73	**<.001**
**Education**									
No post-16 qualifications	—	—		—	—		—	—	
Post-16 qualifications	0.64	0.62, 0.66	**<.001**	0.96	0.93, 0.98	**.001**	0.71	0.68, 0.75	**<.001**
Higher education	0.39	0.38, 0.40	**<.001**	1.03	1.01, 1.05	**.004**	0.49	0.47, 0.50	**<.001**
**Country**									
United Kingdom	—	—		—	—		—	—	
Australia	1.39	1.23, 1.56	**<.001**	0.77	0.70, 0.85	**<.001**	1.26	1.07, 1.47	**.004**
Canada	1.53	1.37, 1.69	**<.001**	0.65	0.60, 0.71	**<.001**	1.09	0.93, 1.26	.3
India	1.29	1.13, 1.46	**<.001**	0.07	0.06, 0.09	**<.001**	0.40	0.31, 0.51	**<.001**
Ireland	2.64	2.47, 2.82	**<.001**	1.68	1.59, 1.78	**<.001**	2.97	2.74, 3.21	**<.001**
New Zealand	1.08	0.92, 1.26	.4	0.74	0.66, 0.83	**<.001**	1.12	0.90, 1.37	.3
Other	2.01	1.93, 2.10	**<.001**	0.42	0.40, 0.44	**<.001**	1.25	1.17, 1.33	**<.001**
The Netherlands	1.89	1.64, 2.18	**<.001**	0.76	0.67, 0.86	**<.001**	1.38	1.12, 1.69	**.002**
United States	1.77	1.69, 1.86	**<.001**	0.52	0.50, 0.55	**<.001**	1.24	1.17, 1.33	**<.001**

*Note.* OR = odds ratio; CI = confidence interval.

### Stratified analyses

In stratified analyses to explore the significant two-way interactions between high extraversion and low conscientiousness (including all covariates but none of the interaction terms from the previous models), the association of high extraversion with smoking was more pronounced in those with high (OR _adj_ = 1.51, 95% CI = 1.46, 1.56, *p* < .001) compared with low conscientiousness (OR _adj_ = 1.38, 95% CI = 1.35, 1.42, *p* < .001). In contrast, the association of high extraversion with excessive drinking was more pronounced in those with low (OR _adj_ = 1.70, 95% CI = 1.67, 1.74, *p* < .001) compared with high conscientiousness (OR _adj_ = 1.60, 95% CI = 1.56, 1.63, *p* < .001). Finally, the association of high extraversion with both behaviours combined was more pronounced in those with high (OR _adj_ = 1.74, 95% CI = 1.65, 1.83, *p* < .001) compared with low conscientiousness (OR _adj_ = 1.62, 95% CI = 1.56, 1.68, *p* < .001).

### Bayes factors

The calculation of Bayes Factors (BFs) indicated that the data on the three-way interactive effect of extraversion, conscientiousness and neuroticism on smoking and excessive drinking provided evidence for the null hypothesis of there not being any effect compared with large associations of OR = 1.4 (BF = 0.07 and BF = 0.03, respectively).

### Sensitivity analyses

The results remained largely robust in the planned sensitivity analysis in which respondents who scored 1 SD above the mean POMP score for each trait were categorised as ‘high’ (or 1 SD below the mean as ‘low’ for conscientiousness), with the remaining respondents categorised as ‘low’ (or ‘high’ for conscientiousness). As in the primary analysis, no significant three-way interactions were observed. In the covariate adjusted model, there were similar main effects and a significant two-way interactive effect of high extraversion and low conscientiousness on excessive drinking (see the Supplementary Online Information, Supplementary Table 1; https://osf.io/c6vmr/). However, unlike the primary analysis, no such two-way interactive effect was observed for smoking, while a two-way interactive effect of low conscientiousness with high neuroticism reached significance, as did a two-way interactive effect of high extraversion with high neuroticism on excessive drinking.

In the unplanned sensitivity analysis, excluding participants falling within 1 SD of the mean on the selected personality traits, there were fewer significant associations, but in the covariate adjusted model, the three-way interactive effect of high extraversion, low conscientiousness and high neuroticism on excessive drinking reached significance (see the Supplementary Online Information, Supplementary Table 2). In stratified analyses to explore the significant three-way interaction, the association of low conscientiousness with excessive drinking was more pronounced in those with low neuroticism and high extraversion (OR _adj_ = 2.25, 95% CI = 2.02, 2.51, *p* < .001) compared with those with high neuroticism and high extraversion (OR _adj_ = 1.87, 95% CI = 1.50, 2.32, *p* < .001), high neuroticism and low extraversion (OR _adj_ = 1.87, 95% CI = 1.65, 2.14, *p* < .001) and low neuroticism and low extraversion (OR _adj_ = 1.41, 95% CI = 1.10, 1.80, *p* = .006).

## Discussion

### Principal findings

This study identified two-way combinations of personality traits (‘personality typologies’) that were associated with greater odds of being a smoker, excessive drinker or both. Specifically, we observed a two-way interactive effect of extraversion and conscientiousness on smoking, excessive drinking and both behaviours combined. The magnitude (but not direction) of the effects differed depending on the level of conscientiousness. However, as results were only somewhat robust in two sensitivity analyses with different methods of categorising respondents into the levels of the exposure variables, these results must be interpreted with caution. Contrary to expectations, no significant three-way interactions were observed in the primary analysis. The calculation of Bayes Factors indicated that data provided evidence for the null compared with large associations for smoking and excessive drinking. Our findings are somewhat at odds with those reported in the literature: for example, the combination of low conscientiousness and high neuroticism has previously been associated with increased odds of being a smoker ( Terracciano & Costa Jr, 2004). Indeed, this was replicated in the first (but not the second) sensitivity analysis with a slightly different method of categorising respondents into the levels of the exposure variables.

### Strengths and limitations

To our knowledge, this was the first study to examine whether ‘personality typologies’ are synergistically associated with greater odds of being a smoker, excessive drinker or both. This study was further strengthened by the large sample size and international recruitment. However, this study also had several limitations. First, the generalisability of the findings is likely limited due to self-selection bias. Although the UK proportion of the sample has previously been found to be representative of the UK population with regards to local authority districts, age and ethnicity ( Rentfrow *et al.*, 2015), our analytic sample was biased towards women, respondents with high education and the proportion of current smokers was substantially lower than what would have been expected from representative population surveys (e.g. 20% of adults aged 16+ years in England in 2012). However, the focus of the study was to examine associations between variables of interest (as opposed to prevalence of specific characteristics) and we deem it unlikely that respondents with a specific combination of personality traits and behaviour(s) have self-selected out of the sample. Second, the BBC Lab UK database does not include validated measures of nicotine dependence or patterns of alcohol consumption, such as the Heaviness of Smoking Index ( Heatherton *et al.*, 1989) or the Alcohol Use Disorders Identification Test ( Babor *et al.*, 2001). This may limit comparisons of the results from this study with those in the wider literature. However, it should be noted that observed associations of sociodemographic characteristics and smoking/excessive drinking were in line with the extant literature. Third, although contested ( Jokela *et al.*, 2018), health behaviours such as smoking and drinking may themselves influence the stability and change of personality over time ( Allen *et al.*, 2015; Stephan *et al.*, 2019). Due to the cross-sectional nature of the study, we were unable to explore potential temporal trends in personality. Fourth, data collection for the BBC Lab UK Study ceased in 2013. Although the prevalence of smoking and excessive drinking has changed, we deem it unlikely that the associations under investigation have changed over time.

### Implications for policy and practice

The finding that respondents with certain combinations of personality traits have greater odds of being a smoker, excessive drinker or both may be useful for informing the tailoring of behaviour change interventions to better engage individuals with high-risk personality typologies. As the identification of at-risk individuals does not require anything other than a brief self-report instrument, this form of tailoring may be particularly feasible and attractive for practitioners to implement. However, as the results produced based on the median split were not completely robust in the two sensitivity analyses (with the exception of high extraversion and low conscientiousness being associated with excessive drinking), additional work is required to establish meaningful cut-offs for the personality traits, particularly in the absence of population-level trait norms. A useful approach for future work may be to select an analytic technique that maximises variance explained, automatically selecting different cut-offs in relation to smoking and excessive alcohol consumption (e.g. principal components analysis or estimating a receiver operating characteristic curve) and analyses should be repeated were population-level norms to become available.

## Conclusion

We identified two-way ‘personality typologies’ that are associated with greater odds of smoking, excessive drinking and both behaviours combined. The results may be useful for the tailoring of behaviour change interventions to at-risk individuals.

## Data availability

### Underlying data

We used data from the BBC Lab UK Study for this work. We do not have approval to release the individual-level data underpinning the analyses. Anonymised and de-identified individual-level data are available upon request from the corresponding author to bona fide researchers and following approval from the British Broadcasting Corporation (BBC).

### Extended data

Analysis code available from: https://github.com/OlgaPerski/Personality-typologies

Archived analysis code as at time of publication: https://doi.org/10.5281/zenodo.5862139 ( Perski, 2022).

This project contains the following files:

- personality_smoking_drinking_28.04.21.R (source code used for analysis)
- variable_names.R

License: MIT
