# Supplementary material for: Personality typologies of smokers and excessive drinkers: a cross-sectional survey of respondents in the BBC Lab UK Study
Source: F1000Res. 2024 Jan 26;11:94. Originally published 2022 Jan 26. [Version 3] doi: 10.12688/f1000research.86670.3 (PMC10690036; doi:10.12688/f1000research.86670.3)
Supplement: Peer review history

# Reviewer report: Reviewer response for version 3

**DOI:** 10.5256/f1000research.160066.r241033

**Published:** 2024-02-09

**Authors:** Jaron Hoani King

**Affiliations:** 1 Department of Health Promotion, Education, and Behavior, University of South Carolina, Columbia, South Carolina, USA

Wonderful work. I have no further comments.

Is the work clearly and accurately presented and does it cite the current literature?

Yes

If applicable, is the statistical analysis and its interpretation appropriate?

Yes

Are all the source data underlying the results available to ensure full reproducibility?

Yes

Is the study design appropriate and is the work technically sound?

Yes

Are the conclusions drawn adequately supported by the results?

Yes

Are sufficient details of methods and analysis provided to allow replication by others?

Yes

Reviewer Expertise:

Public Health (substance use)

I confirm that I have read this submission and believe that I have an appropriate level of expertise to confirm that it is of an acceptable scientific standard.

# Reviewer report: Reviewer response for version 2

**DOI:** 10.5256/f1000research.144887.r226597

**Published:** 2023-11-30

**Authors:** Jaron Hoani King

**Affiliations:** 1 Department of Health Promotion, Education, and Behavior, University of South Carolina, Columbia, South Carolina, USA

This article is a fascinating attempt to identify if any relationships exist between the "Big Five" personality model and certain health risk behaviors or profiles, namely cigarette smoking and excessive alcohol consumption. All items on the peer review form were satisfied. Below is a review for the authors to consider in future work:

 In many cultures around the world, smoking and excessive drinking are socially undesirable. As a result, it has been noted that personalities can both inform behavior (the decision to first engage in an addictive behavior) and behavior can actually change personality (as noted by the authors in the limitations section). When considering the potential synergistic effect, would it be necessary to "score" individuals on all 5 personality traits? To my understanding, the analysis here essentially set aside the other two personality traits that were not analyzed ("openness to experience" and "agreeableness"). Because everyone would score somewhere along the spectrum for all five personality traits, would it not be important to consider all five traits at once? If you limited the analytic sample to only those who were within 1 SD of the median for "openness to experience" and "agreeableness" would your results significantly change? It seems plausible that one or both of these traits act as a sort of mediator with the synergistic trio you hypothesized: low conscientiousness, high extraversion, and high neuroticism (e.g., if someone had this personality typology coupled with high agreeableness they may be more susceptible to influence by social norms than those with low agreeableness).

 While a formal analysis may not be necessary, it may be helpful context to describe in the discussion section why other variations *within *the described three-way typology (e.g., those with high agreeableness vs. those with low agreeableness) were not considered. Outside of this suggestion to consider if the combination results of the two non-studied typologies *may* impact results, I have no other comments.

 Thank you for the opportunity and for sharing this interesting work.

Is the work clearly and accurately presented and does it cite the current literature?

Yes

If applicable, is the statistical analysis and its interpretation appropriate?

Yes

Are all the source data underlying the results available to ensure full reproducibility?

Yes

Is the study design appropriate and is the work technically sound?

Yes

Are the conclusions drawn adequately supported by the results?

Yes

Are sufficient details of methods and analysis provided to allow replication by others?

Yes

Reviewer Expertise:

Public Health (substance use)

I confirm that I have read this submission and believe that I have an appropriate level of expertise to confirm that it is of an acceptable scientific standard.

# Reviewer report: Reviewer response for version 2

**DOI:** 10.5256/f1000research.144887.r165258

**Published:** 2023-03-21

**Authors:** Arielle S. Selya

**Affiliations:** 1 Pinney Associates, Inc., Pittsburgh, PA, USA

I thank the authors for responding to my comments. I have no further suggestions.

Is the work clearly and accurately presented and does it cite the current literature?

Yes

If applicable, is the statistical analysis and its interpretation appropriate?

Yes

Are all the source data underlying the results available to ensure full reproducibility?

Partly

Is the study design appropriate and is the work technically sound?

Yes

Are the conclusions drawn adequately supported by the results?

Yes

Are sufficient details of methods and analysis provided to allow replication by others?

Yes

Reviewer Expertise:

Tobacco use behavior

I confirm that I have read this submission and believe that I have an appropriate level of expertise to confirm that it is of an acceptable scientific standard.

# Reviewer report: Reviewer response for version 1

**DOI:** 10.5256/f1000research.92008.r157918

**Published:** 2023-01-27

**Authors:** Arielle S. Selya

**Affiliations:** 1 Pinney Associates, Inc., Pittsburgh, PA, USA

This manuscript examines the interactive effects of personality dimensions on outcomes of smoking, excessive drinking, and both behaviors together, using data from the BBC Lab UK Study, with the hypothesis being that the combination of low conscientiousness, high extraversion, and high neuroticism would interact to have higher risk of smoking and drinking. Results instead found that the combination of high extraversion and *high* conscientiousness was associated with two of the outcomes (smoking and both behaviors combined), while the combination of high extraversion and low conscientiousness was associated with excessive drinking.

**Major comments:**

- The Introduction appropriately reviews the five major dimensions of personality, but the hypothesis focuses only on three of them. Why were these particular aspects chosen?
- The authors explain that there are currently no population norms for personality dimensions. How does this impact the implications that these dimensions might be used to identify at-risk individuals?

**Minor comments:**

- Typo in Introduction/conscientiousness: “non0pt-representative”.
- What was the rationale behind the deviation from the pre-registration with respect to the categorization of “excessive drinking?”
- Table 1 lists the number excluded from the sample (N=224,560) but the exclusion criteria do not seem to be explained in the text – please add

Is the work clearly and accurately presented and does it cite the current literature?

Yes

If applicable, is the statistical analysis and its interpretation appropriate?

Yes

Are all the source data underlying the results available to ensure full reproducibility?

Partly

Is the study design appropriate and is the work technically sound?

Yes

Are the conclusions drawn adequately supported by the results?

Yes

Are sufficient details of methods and analysis provided to allow replication by others?

Yes

Reviewer Expertise:

Tobacco use behavior

I confirm that I have read this submission and believe that I have an appropriate level of expertise to confirm that it is of an acceptable scientific standard, however I have significant reservations, as outlined above.

# Response

**Published:** 2023-02-22

**Authors:** Olga Perski

**Affiliations:** University College London, UK

*Thank you for the helpful feedback on our manuscript. Please find point-by-point responses to the comments below.*

**Major comments:**

 The Introduction appropriately reviews the five major dimensions of personality, but the hypothesis focuses only on three of them. Why were these particular aspects chosen?

*Thank you – we have now added the following explanation to the end of the Introduction:*

*“As limited research has been conducted relating to openness to experience and agreeableness, with available studies providing mixed evidence of the associations between these personality traits and addictive behaviours, we were unable to generate a directional hypothesis for these traits.”*

 The authors explain that there are currently no population norms for personality dimensions. How does this impact the implications that these dimensions might be used to identify at-risk individuals?

*We now further reflect on this in the Discussion:*

*“As this study involved a very large sample, using the median split is likely to provide initially useful population-level insight as to what cut-off may be useful for identifying at-risk individuals. However, although such a pragmatic approach provides initial utility in the absence of population-level norms, further work is required to establish meaningful cut-offs and analyses should be repeated were population-level norms to become available.”*

**Minor comments:**

 Typo in Introduction/conscientiousness: “non0pt-representative”.

*This has now been amended.*

 What was the rationale behind the deviation from the pre-registration with respect to the categorization of “excessive drinking?”

*We now provide additional explanation in the Methods:*

*“This deviation was based on a better fit between our categorisation and a validated measure (AUDIT-C). The original categorisation would have overestimated excessive drinking.”*

 Table 1 lists the number excluded from the sample (N=224,560) but the exclusion criteria do not seem to be explained in the text – please add

*We already state that respondents with missing data on any of the variables of interest were excluded from the analyses. There were no additional exclusion criteria.*
